# Supplementary material for: Acupuncture on ST36, CV4 and KI1 Suppresses the Progression of Methionine- and Choline-Deficient Diet-Induced Nonalcoholic Fatty Liver Disease in Mice
Source: Metabolites. 2019 Dec 9;9(12):299. doi: 10.3390/metabo9120299 (PMC6949943; doi:10.3390/metabo9120299)
Supplement: Supplementary file 1 [file metabolites-09-00299-s001.pdf]

Supplementary materials

Acupuncture on ST36, CV4 and KI1 suppresses the progression of methionine- and choline deficient diet-induced nonalcoholic fatty liver disease in mice

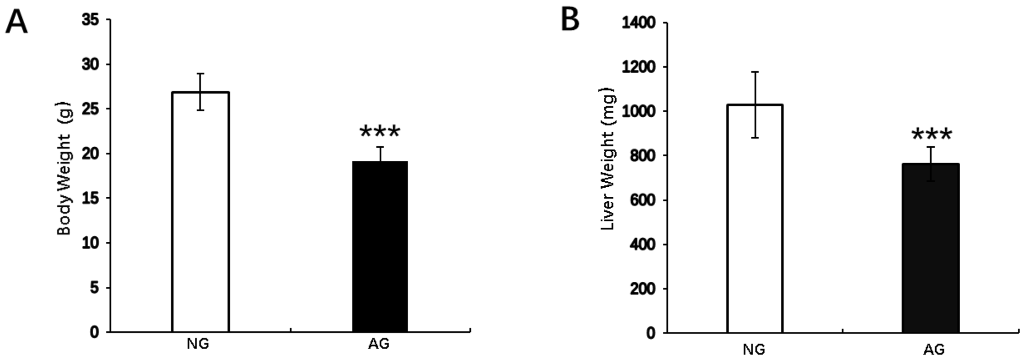

Figure S1. The body weight and liver weight after two weeks of acupuncture. (A) The body weight was significantly lower in AG mice than in those of NG mice. Values are shown as the mean ± SD, *p<0.05, **p<0.001, ***p<0.0001, n=17. (B) The liver weight was significantly lower in AG mice than in those of NG mice. Values are shown as the mean ± SD, *p<0.05, **p<0.001, ***p<0.0001, n=17. NG: needling-nonacupoint group, AG: needling-acupoint group, MCD diet: methionine- and choline-deficient diet, HF diet: high-fat diet, NAFLD: nonalcoholic fatty liver disease.

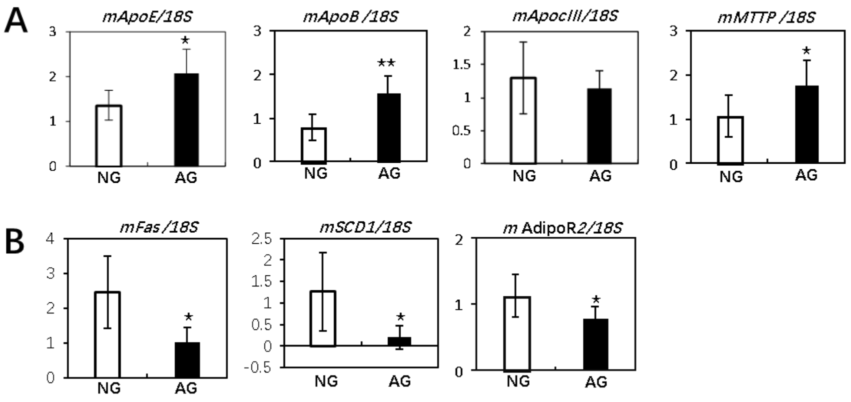

Figure S2. Acupuncture improved the lipid metabolism in the livers of mice with NAFLD. (A) Real-time-PCR revealed that the expression of ApoB, ApoE and MTTP were significantly higher in the livers of AG mice than in those of NG mice. ApoC-III showed no significant difference between the groups. (B)The expression of Fas, SCD1 and AdipoR2 were significantly lower in the livers of AG mice than in those of NG mice. Values are normalized by the 18S rRNA expression. RT-PCR values are presented as the means ± SD. *$p<0.05$, **$p<0.001$. ***$p<0.0001$, **n=7**. NG: needling-nonacupoint group, AG: needling-acupoint group, ApoB: apolipoprotein B, ApoE: apolipoprotein E, MTTP: microsomal triglyceride transfer protein, ApoC-III: apolipoprotein C-III, Fas: fat acid syntheas, SCD1: stearoyl-CoA 9-desaturase 1, AdipoR2: adiponectin receptor 2.
